# Prediction uncertainty estimates elucidate the limitation of current NSCLC subtype classification in representing mutational heterogeneity

**DOI:** 10.1038/s41598-024-57057-3

**Published:** 2024-03-21

**Authors:** Andrei Puiu, Carlos Gómez Tapia, Maximilian E. R. Weiss, Vivek Singh, Ali Kamen, Matthias Siebert

**Affiliations:** 1 Advanta, Siemens SRL, Brasov, 500007 Romania; 2 Automation and Information Technology, Transilvania University of Brasov, Brasov, 500174 Romania; 3 Digital Technology and Innovation, Siemens Healthineers, Erlangen, 91052 Germany; 4 Digital Technology and Innovation, Siemens Healthineers, Princeton, 08540 USA

**Keywords:** Non-small-cell lung cancer, Machine learning, Cancer genomics

## Abstract

The heterogeneous pathogenesis and treatment response of non-small cell lung cancer (NSCLC) has led clinical treatment decisions to be guided by NSCLC subtypes, with lung adenocarcinoma and lung squamous cell carcinoma being the most common subtypes. While histology-based subtyping remains challenging, NSCLC subtypes were found to be distinct at the transcriptomic level. However, unlike genomic alterations, gene expression is generally not assessed in clinical routine. Since subtyping of NSCLC has remained elusive using mutational data, we aimed at developing a neural network model that simultaneously learns from adenocarcinoma and squamous cell carcinoma samples of other tissue types and is regularized using a neural network model trained from gene expression data. While substructures of the expression-based manifold were captured in the mutation-based manifold, NSCLC classification accuracy did not significantly improve. However, performance was increased when rejecting inconclusive samples using an ensemble-based approach capturing prediction uncertainty. Importantly, SHAP analysis of misclassified samples identified co-occurring mutations indicative of both NSCLC subtypes, questioning the current NSCLC subtype classification to adequately represent inherent mutational heterogeneity. Since our model captures mutational patterns linked to clinical heterogeneity, we anticipate it to be suited as foundational model of genomic data for clinically relevant prognostic or predictive downstream tasks.

## Introduction

Next-generation sequencing (NGS)-based genotyping technologies are increasingly being employed to support clinical decision-making. The standard approach to developing diagnostic, prognostic, or predictive models based on NGS-generated high-dimensional data is to preselect a small number of biomarkers, e.g., gene mutations that are identified to be independently associated with the phenotype of interest, and restrict training of a basic statistical model to this restricted set of biomarkers, e.g., to predict survival benefit from immunotherapies^1,2^.

In fact, disease etiology and treatment response are often complex, affected by a multitude of genomic alterations, each exerting a small effect on the phenotype. Therefore, it is expected that the consideration of all identified alterations broadens the range of phenotype risk. However, due to the high dimensionality of genomic data, inherent genetic heterogeneity, as well as small patient cohorts, the robust training of predictive models is challenging^3^.

Functional readouts of cellular activity and physiological status as provided by omics technologies such as RNA sequencing, measuring genome-wide changes in mRNA expression, are expected to have greater capacity to inform clinical management decisions, including diagnosis, prognosis, treatment selection, and monitoring. Ideally, omics technologies that capture the complex molecular interplay within and across different biological levels should be combined^4^. Still, due to practical and financial reasons, genomics is the most clinically adopted omics modality to date^4–7^.

In cancer, histology-based classification of tumors reflects different clinical presentation and course of the disease. For instance, lung cancers are classified as small-cell (SCLC) or non-small cell (NSCLC). In addition, NSCLCs are further subdivided into subtypes such as lung adenocarcinoma (LUAD) and lung squamous cell carcinoma (LUSC). While morphology-based determination may be inconclusive, LUADs and LUSCs show distinct genetic drivers and cellular signaling activities^8^, influenced by the cell type of origin^9^.

Importantly, different prognostic determinants have been identified in LUAD vs. LUSC, which also partly show opposite impact on clinical outcome^10^. In addition, NSCLC histology was found to be predictive of treatment response. For instance, platinum-based adjuvant chemotherapy for patients with completely resected early-stage NSCLC conferred a survival benefit in LUSCs but not LUAD^11^. In contrast, pemetrexed chemotherapy only showed improved efficacy compared to other standard treatment options in patients with advanced non-squamous NSCLC^12^. NSCLC histology is also considered predictive of response to targeted therapies and immunotherapies in the latest ASCO guidelines for late-stage NSCLC patients with^13^ and without^14^ actionable driver alterations. The combined treatment of NSCLC with chemotherapy and immunotherapy is the subject of ongoing investigations as part of clinical trials^15^.

Analogous to the differential response to anticancer drugs, the dose effect of stereotactic body radiation therapy (SBRT) has also been described to differ between NSCLC subtypes^16,17^, with an increased local failure rate in patients with LUSC, indicating the need for histology-specific treatment adjustment. Therefore, knowledge of NSCLC histology is essential in the optimized selection from available therapy options.

In this work, we aimed at improving NSCLC subtype classification from mutational data and developed a deep genomic profiling model that, in addition to LUAD and LUSC samples, simultaneously learns from adenocarcinoma (AD) and squamous cell carcinoma (SCC) samples of other tissue types and that is regularized using a neural network model trained from gene expression data. Notably, classification performance can be improved on samples with confident predictions, identified with an ensemble approach capturing prediction uncertainties. Moreover, uncertainty estimates of misclassified samples indicate limitations of the current NSCLC classification scheme in representing mutational heterogeneity within subtypes, potentially impeding the prediction of treatment outcome.

## Results

**Figure 1:**
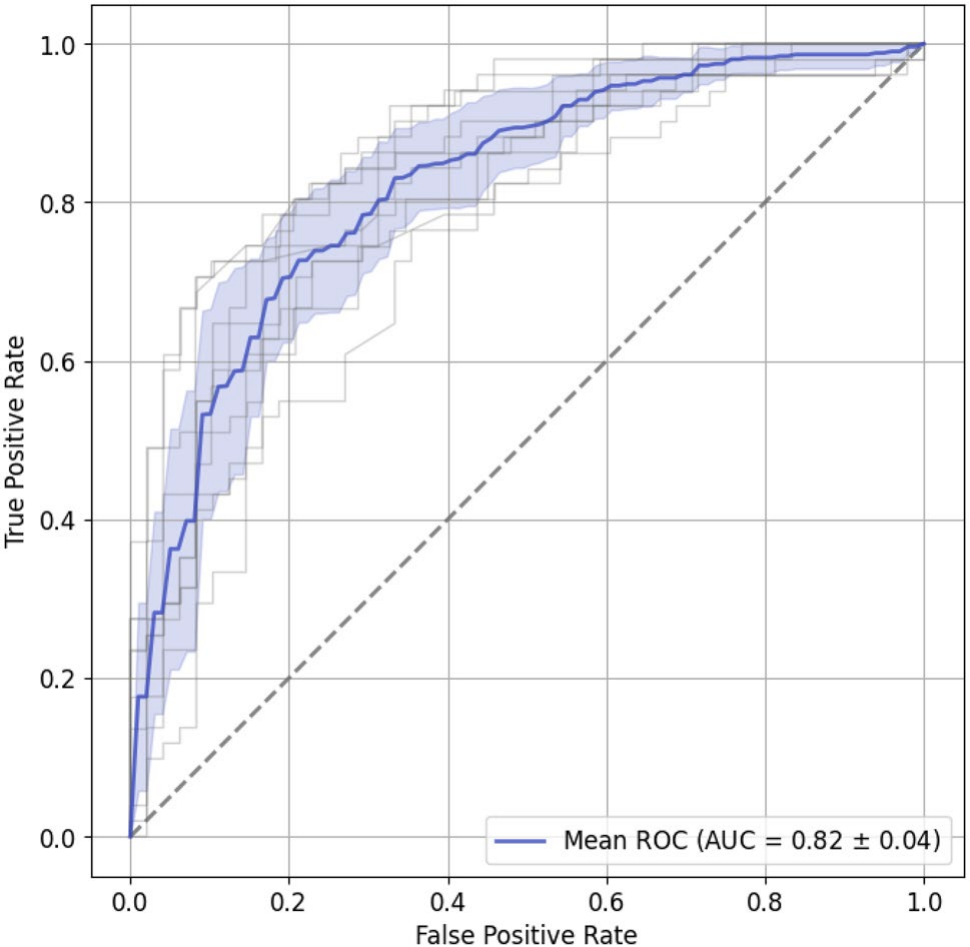
Performance of the baseline genomic profiling model to classify NSCLC subtypes using mutational data. The blue line and light blue area depict the mean ROC curve and its standard deviation, respectively, across ten cross-validation folds. Grey lines correspond to ROC curves of individual folds. The dashed line represents the ROC curve of a random classifier.

To establish a baseline, we trained a genomic profiling model consisting of a multilayer perceptron (MLP) to classify NSCLC samples into LUAD and LUSC subtypes using mutational data. This baseline model achieved an area under the receiver operating characteristic (ROC) curve (AUC) of 0.82 as a measure of classification performance on test samples using a tenfold cross-validation scheme (Fig. 1).

### Expression-based regularization improves the manifold, without improving classification performance

**Figure 2:**
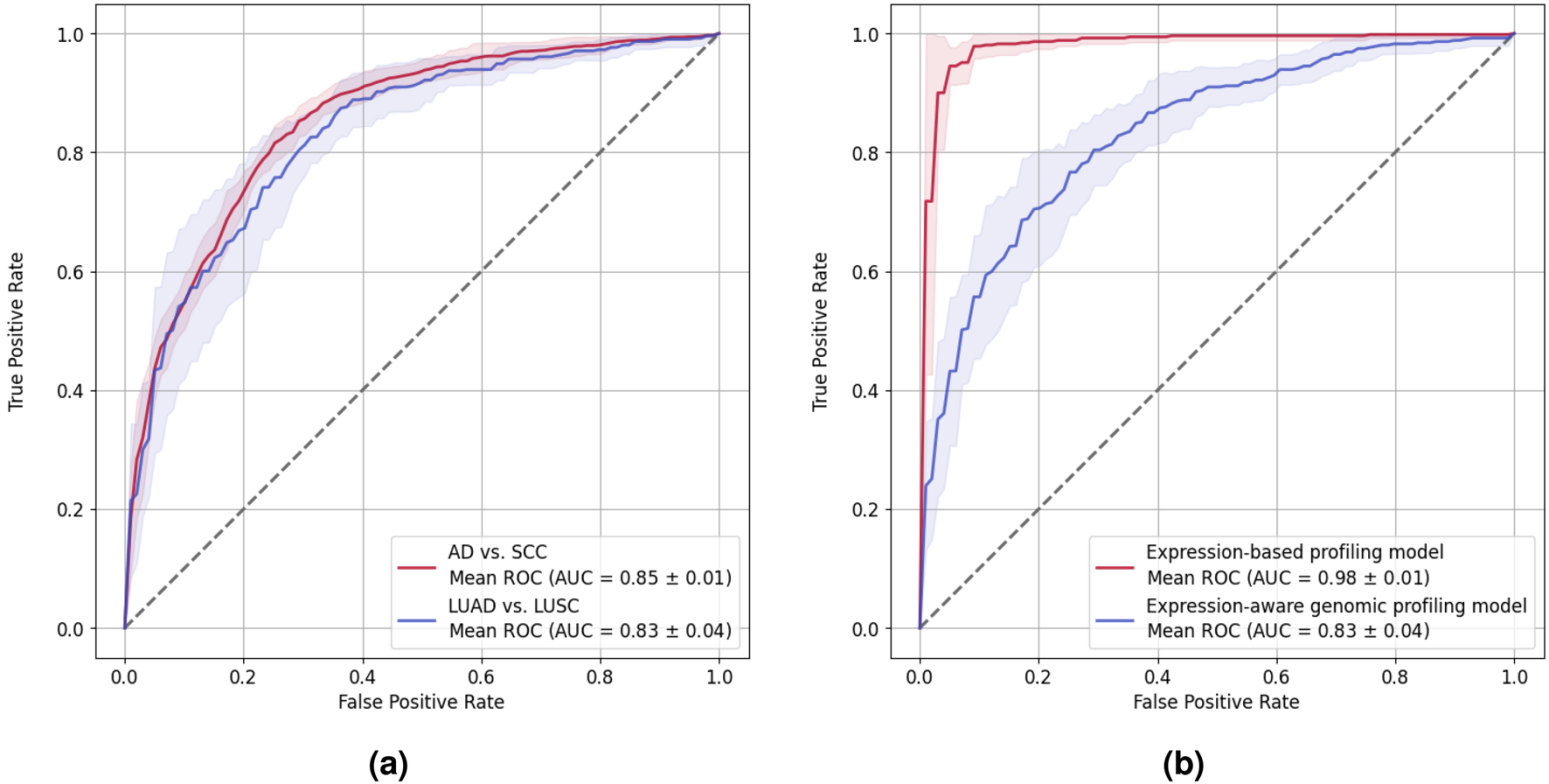
Impact of different regularization approaches on classification performance. (**a**) Performance of the genomic profiling model trained on an extended dataset, including AD and SCC subtypes of additional non-NSCLC cancer types. The red curve shows the performance on all samples (using a model with a single prediction head). The blue curve depicts the performance on the subset of NSCLC samples, obtained with the NSCLC-specific prediction head (using a model with two prediction heads). (**b**) Comparison of the performance of the expression-based profiling model (red) to the expression-aware genomic profiling model (blue) in classifying NSCLC subtypes. The light red and blue areas correspond to one standard deviation of the respective mean ROC curves. The dashed lines depict the ROC curve of a random classifier.

The classification performance of the baseline genomic profiling model could not be exceeded, irrespective of different training and model parameter configurations tested, demonstrating the challenge this sparse, high-dimensional genomic dataset presents. To overcome this challenge, we created an extended dataset by augmenting the NSCLC dataset with additional AD and SCC samples of non-NSCLC histology. This extended dataset served as a regularizer by training a genomic profiling model to simultaneously classify AD and SCC samples of the lung as well as other tissue types using two prediction heads. However, the classification performance on NSCLC samples did not significantly improve over the baseline genomic profiling model (Fig. 2a, blue ROC curve), notwithstanding that the genomic profiling model with one prediction head achieved an AUC of 0.85 (± 0.01) in classifying samples from all tissue types into AD vs. SCC histology (Fig. 2a, red ROC curve).

**Figure 3:**
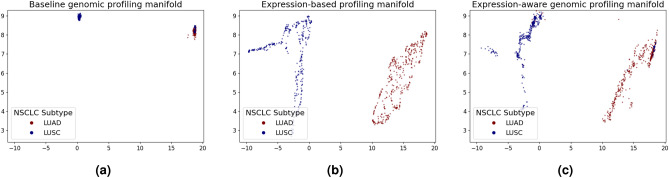
2D projections of the manifold learned with (**a**) the baseline genomic profiling model, (**b**) the expression-based profiling model, and (**c**) the expression-aware genomic profiling model, obtained with UMAP using a local neighborhood of size 15. Each point corresponds to either a LUAD (red) or a LUSC (blue) sample.

Since NSCLC subtypes were found to be distinct at the transcriptomic level^8^, we further aimed at improving NSCLC classification accuracy by regularizing training of the genomic profiling model with the latent representation learned by a gene expression-based profiling model, thereby obtaining an expression-aware genomic profiling model. Like previous reports, Fig. 2b shows the outstanding performance of an expression-based profiling model in classifying NSCLC subtypes (red ROC curve), achieving an AUC of 0.98 (± 0.01). Moreover, substructures of the manifold learned by the expression-based profiling model (Fig. 3b) were captured in the expression-aware genomic profiling manifold (Fig. 3c), qualitatively improving over the manifold learned by the baseline genomic profiling model (Fig. 3a). However, regularized training did not succeed in leveraging the prediction capacity of the expression-based profiling model to improve the classification performance of the expression-aware over the baseline genomic profiling model (Fig. 2b, blue ROC curve).

### Prediction uncertainty estimates enable increased performance on confident samples

The results indicate an inherent complexity and ambiguity in terms of how the genomic profile translates into cellular activity and physiological status. Capturing this uncertainty within the model might allow ambiguous samples to be identified and rejected, while improving the performance on remaining samples. To this end, we employed a bootstrap aggregating (also called bagging) approach to train an ensemble of one hundred expression-aware genomic profiling models and calculated an aggregated prediction score by averaging prediction values of all models predicting the majority predicted NSCLC subtype.

**Figure 4:**
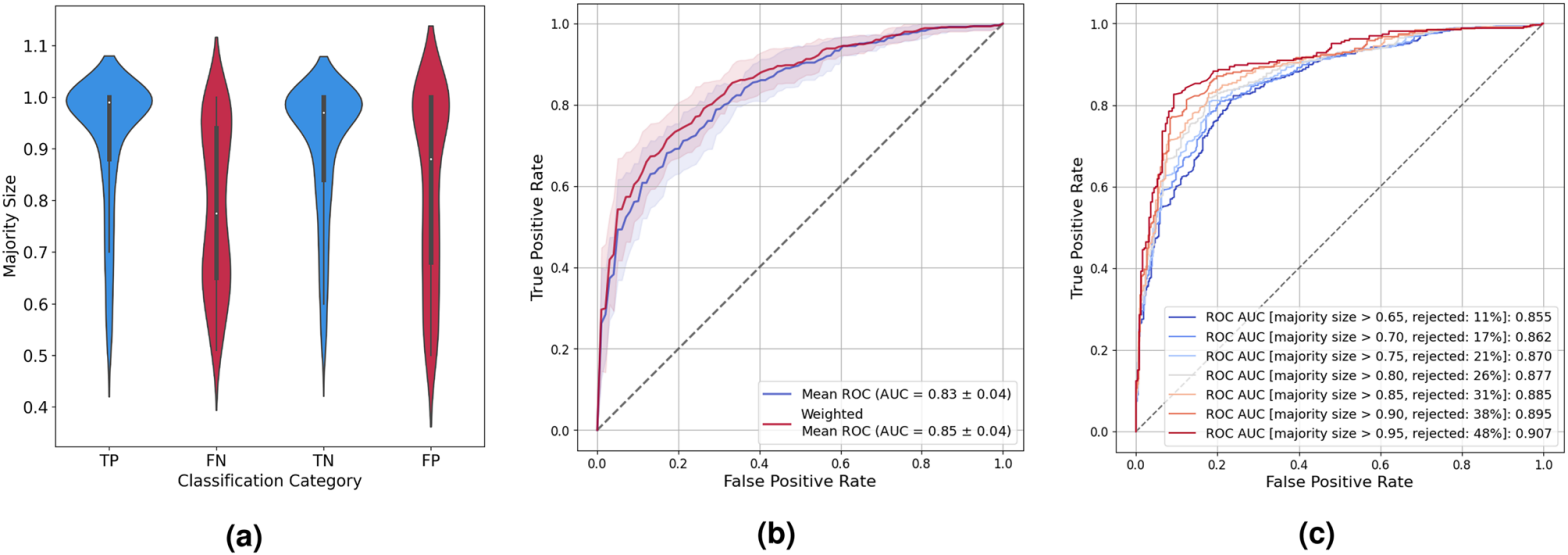
Performance of an ensemble of expression-aware genomic profiling models trained to classify NSCLC subtypes using mutational data of the extended dataset. (**a**) Distribution of the majority size as uncertainty estimate for different classification categories, with TP, FN, TN, and FP corresponding to true positive, false negative, true negative, and false positive predictions, respectively. (**b**) Performance of the model calculated with (red) and without (blue) uncertainty-based weighting of samples. (**c**) Performance of the model when applying different uncertainty estimate thresholds and rejecting respective test samples. In addition to the applied threshold, the legend lists the fraction of rejected in all samples.

Although the classification performance of the ensemble remained unchanged (Fig. 4b, blue ROC curve), the ensemble enabled inconclusive samples to be identified by calculating the fraction of models predicting the majority predicted NSCLC subtype (termed majority size) as a measure of prediction uncertainty.

Figure 4a shows the agreement between the models of the ensemble separately for each classification category. Since the agreement is greater for correctly classified (TP and TN) as opposed to misclassified (FP and FN) samples, the majority size turned out to be a reasonable measure of prediction uncertainty.

Indeed, the AUC could be increased by 0.02 when utilizing the uncertainty estimates as weights in the calculation of the ROC curve (Fig. 4b, red ROC curve). Besides, a threshold value can be applied to reject samples with high prediction uncertainty and, as a result, increase classification performance on remaining samples, as demonstrated in Fig. 4c. Crucially, the AUC monotonically increased the more restrictive the threshold applied. However, there is a tradeoff between classification performance and the fraction of rejected samples. For instance, imposing a minimum majority size of 0.75 increased the AUC to 0.87 but also implicated the rejection of 21% of the test samples. Likewise, the AUC exceeded 0.9 when applying a more restrictive threshold of 0.95, with the downside that nearly half of the test samples were rejected.

### Conflicting mutational patterns limit classification performance

To get a better understanding of the source of classification errors, we analyzed the correlation of misclassified samples with clinical characteristics, such as age and sex, as well as sample characteristics, such as tumor stage, tumor mutational burden, leukocyte fraction, stromal fraction, intratumor heterogeneity, and immune subtypes^18^. However, we could not uncover any significant association, irrespective of classification category.

To estimate the contribution of each feature, i.e., the mutational status of each gene, to model predictions, we conducted a SHAP (SHapley Additive exPlanations) analysis using KernelSHAP^19^. This local explainability approach trains a surrogate model to learn the Shapley values of different feature combinations (termed coalitions) as its weights and calculates the average contribution of each feature to the predictions of different coalitions in comparison to the average prediction across all samples.

**Figure 5:**
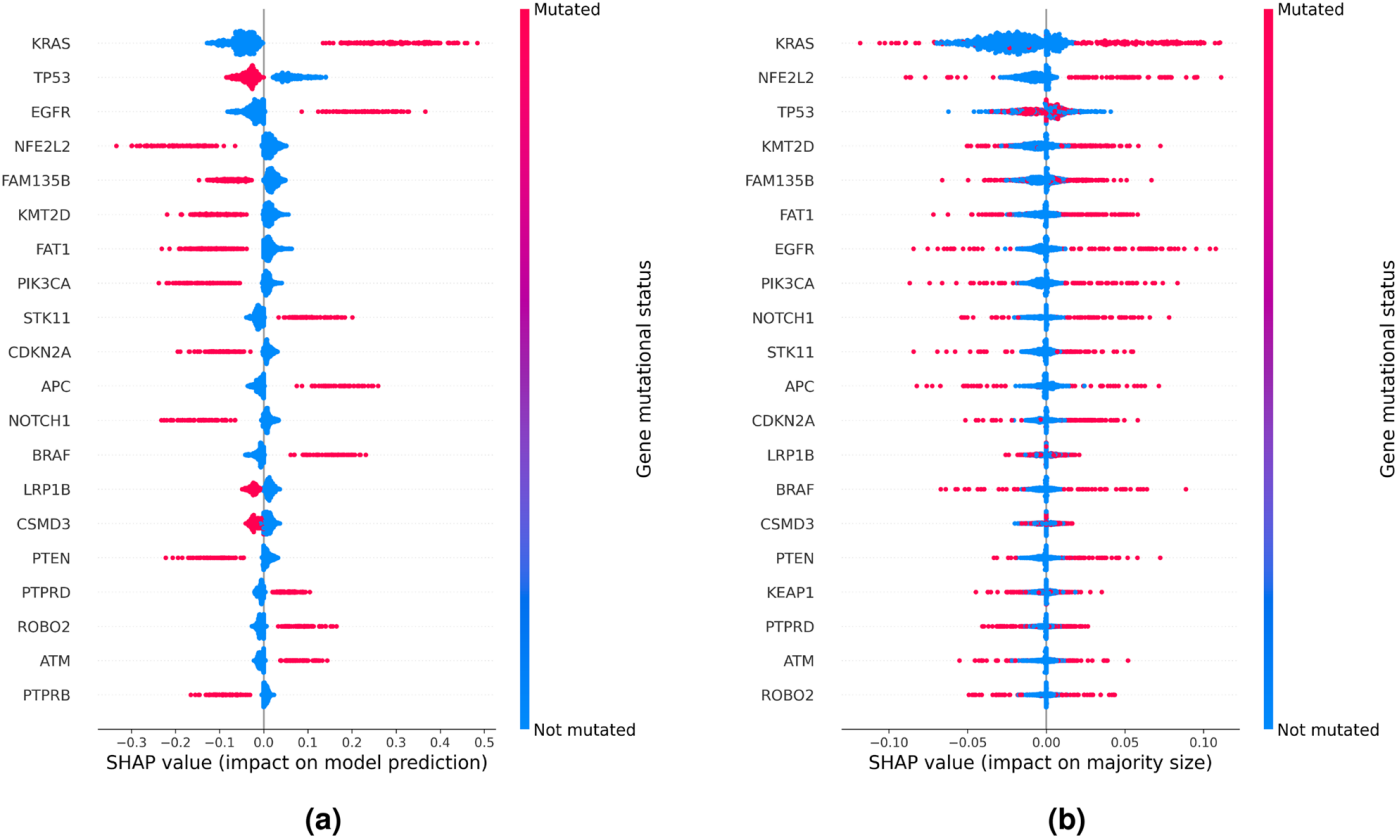
Cohort-level SHAP summaries showing the contribution of the mutational status of the 20 genes with greatest impact on (**a**) model prediction and (**b**) prediction uncertainty. The analysis is based on predictions of the ensemble of expression-aware genomic profiling models trained to classify NSCLC subtypes using mutational data of the extended dataset. (**a**) Positive and negative SHAP values correspond to a positive impact of the respective gene mutational status on classifying a sample as LUAD and LUSC, respectively. (**b**) Positive SHAP values correspond to a positive impact of the respective gene mutational status on prediction confidence, whereas prediction uncertainty is increased in case the gene mutational status is assigned a negative SHAP value.

Figure 5a depicts the cohort-level SHAP summary for all samples of the testing set, representing the importance of the mutational status of respective genes to predictions of the ensemble of expression-aware genomic profiling models trained to classify NSCLC subtypes using mutational data of the extended dataset. Each point corresponds to a Shapley value for a gene and a test set sample, determining its position along the x-axis. Its color illustrates the mutational status of a gene in a sample. Genes are ordered along the y-axis with respect to their overall importance for the prediction. The mutational status of KRAS exhibits the greatest overall importance, with mutated and non-mutated KRAS having a large positive impact on classifying a sample as LUAD and LUSC, respectively. The mutational status of TP53 exhibits opposite effects on model predictions. Additional genes indicative of LUAD when mutated, among the ten genes determined to be most important for NSCLC subtype classification, include EGFR and STK11, while mutated NFE2L2, FAM135B, KMT2D, FAT1, PIK3CA, and CDKN2A are predictive of LUSC.

To gain a better understanding of the classification accuracy on NSCLC subgroups comprising all samples that carry a mutated gene identified in the SHAP analysis, we compared our genomic profiling model to univariable models, which, in the presence of the mutated gene, assign the sample to the NSCLC subtype the mutated gene is mostly associated with, and to the other NSCLC subtype, otherwise (Supplementary Table S1). While univariable models perform best on highly imbalanced subgroups, i.e., subgroups in which the selected gene is strongly associated with one of the subtypes when mutated (e.g., KRAS in LUAD, Supplementary Table S2), our genomic profiling model turned out to be superior in all other cases.

We also estimated the contribution of the mutational status of individual genes to prediction uncertainty (Fig. 5b). Overall, 19 of the 20 most important genes, corresponding to genes that are recurrently mutated in NSCLC, are identical. However, gene mutations can show opposite effects on prediction uncertainty, depending on co-occurring mutations.

**Figure 6:**
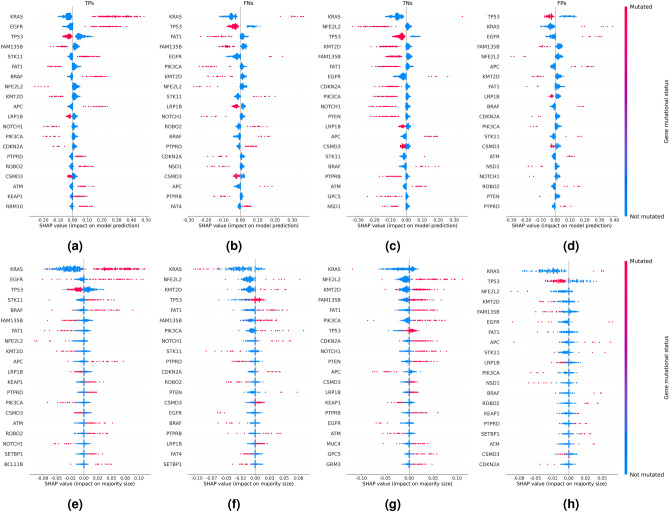
Cohort-level SHAP summaries showing the contribution of the mutational status of the 20 genes with greatest impact on (**a–d**) model prediction and (**e–h**) prediction uncertainty for different prediction categories: (**a,e**) true positive (TP), (**b,f**) false negative (FN), (**c,g**) true negative (TN), and (**d,h**) false positive (FP) predictions, respectively. The analysis is based on predictions of the ensemble of expression-aware genomic profiling models trained to classify NSCLC subtypes using mutational data of the extended dataset.

This effect cannot be detected when contrasting SHAP analyses of correctly to incorrectly classified LUAD samples with respect to model predictions (Fig. 6a,b). However, a SHAP analysis with respect to prediction uncertainties (Fig. 6e,f) reveals that mutations in genes indicative of LUAD, e.g., KRAS, can increase prediction uncertainty due to conflicting mutations in other genes (Fig. 6f). Similar effects can be observed in correctly vs. incorrectly classified LUSC samples (Fig. 6c,d,g,h).

**Figure 7:**
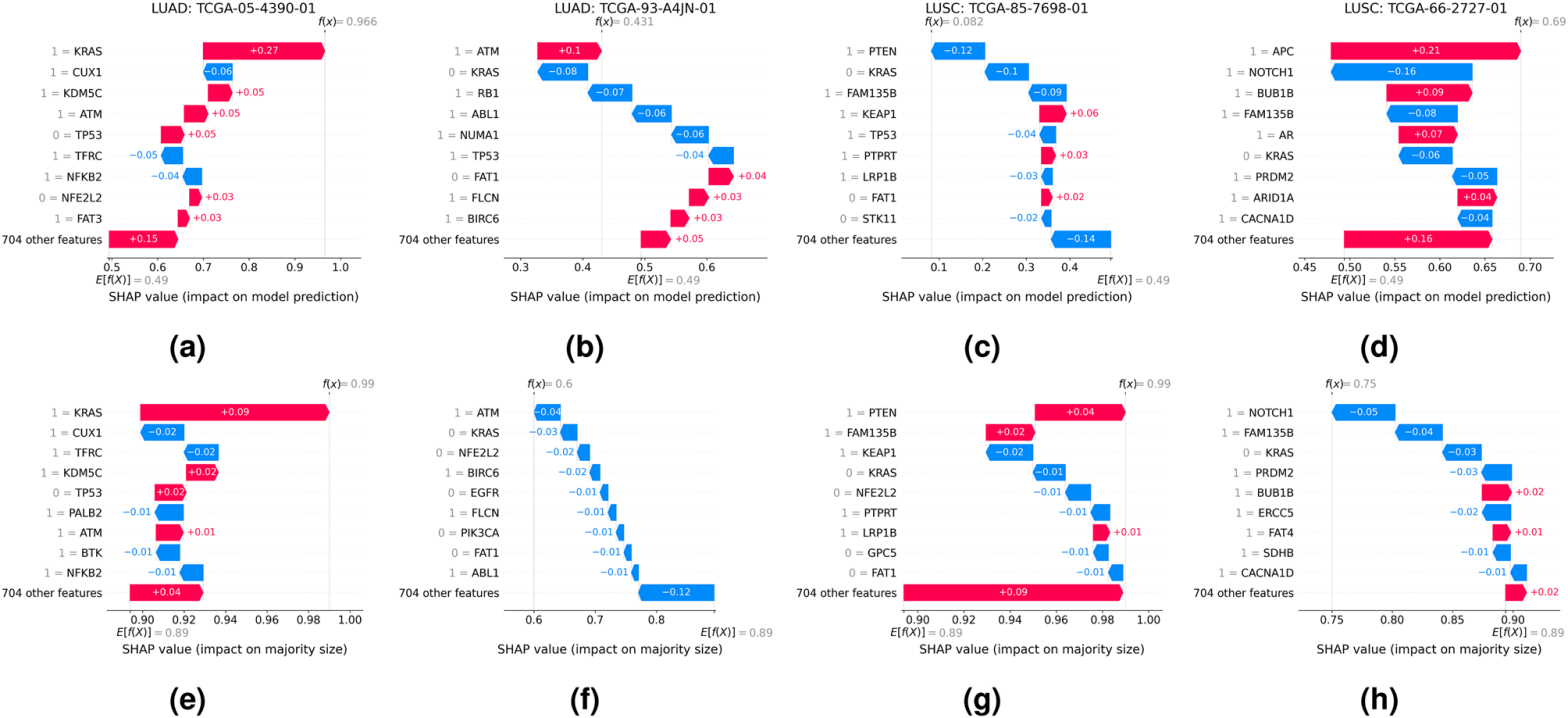
Waterfall plots of SHAP feature attributions showing the contribution of the mutational status of the nine genes with greatest impact on (**a–d**) model prediction and (**e–h**) prediction uncertainty for selected samples, representing (**a,e**) a correctly and (**b,f**) an incorrectly classified LUAD sample with low and high prediction uncertainty, as well as (**c,g**) a correctly and (**d,f**) an incorrectly classified LUSC sample with low and high prediction uncertainty, respectively. f(x) corresponds to (**a–d**) the prediction value (1 = LUAD, 0 = LUSC) and (**e–h**) its associated prediction uncertainty (1 = lowest, 0.5 = highest uncertainty), respectively. Plots are aligned at the average prediction across all test samples, E[(f(x)]. Feature values of 0 and 1 correspond to non-mutated and mutated genes, respectively. Predictions are based on the ensemble of expression-aware genomic profiling models trained to classify NSCLC subtypes using mutational data of the extended dataset.

To explore such mutational patterns on the instance level, the waterfall plots in Fig. 7a,b illustrate the contribution of the mutational status of individual genes to a correct and an incorrect prediction of two selected LUAD samples, respectively. While the mutation in KRAS has a large impact on correctly classifying TCGA-05-4390-01 as a LUAD sample, the inconclusive mutational pattern in LUAD sample TCGA-93-A4JN-01, comprising mutated ATM but also non-mutated KRAS, increases prediction uncertainty (Fig. 7f) due to opposing effects on model prediction (Fig. 7b). In contrast, in LUAD sample TCGA-05-4390-01, mutated ATM decreases prediction uncertainty due to a co-occurring mutation in KRAS (Fig. 7e). Similarly, correct vs. incorrect classifications of LUSC samples can also be attributed to more vs. less consistent mutational patterns, as exemplified with LUSC samples TCGA-85-7698-01 (Fig. 7c,g) and TCGA-66-2727-01 (Fig. 7d,h), respectively.

From these observations, we deduce samples with higher prediction uncertainty to exhibit mutational patterns that are ambiguous with respect to NSCLC histology, which could be indicative of mixed-type histologies. To investigate this further, and due to the lack of a corresponding label (samples are annotated as either LUAD or LUSC), we selected the most confident driver genes for LUAD (BRAF, EGFR, KRAS, and STK11) and LUSC (CDKN2A, NFE2L2, PIK3CA, and PTEN) and show a comparative analysis between samples carrying a mutated LUAD driver, a mutated LUSC driver gene, or both (mixed driver mutations) as a proxy of mixed-type histology. Remarkably, the classification performance of our genomic profiling model is comparable across all subgroups (Supplementary Fig. S1a). Moreover, predictions of mixed driver samples show a trend towards intermediate prediction uncertainty estimates (Supplementary Fig. S1b). Finally, the separation of progression-free survival (PFS) curves underlines the clinical relevance of the mixed driver subgroup (Supplementary Fig. S1c).

## Discussion

The capability of our deep genomic profiling model to assess the confidence in a prediction permits the identification of indeterminate or out-of-distribution samples, which is indispensable for deep learning approaches to become accepted and implemented in clinical practice. In this work, we trained an ensemble of one hundred genomic profiling models. While the size of the ensemble can still be optimized to reduce computing resources, we can also imagine a teacher-student learning setup, providing a distilled model that is more practical for clinical application^20^. Of note, we found the majority size to improve over the prediction variance as a measure of prediction uncertainty (Fig. 4a, Supplementary Fig. S2), presumably as a consequence of the substantial number of samples carrying mutations indicative of both NSCLC subtypes.

Overall, the estimation of prediction uncertainties facilitates the investigation of model predictions, as the contributions of features cannot only be assessed with respect to model predictions but also prediction uncertainties. In fact, such an analysis identified co-occurring mutations indicative of both NSCLC subtypes in misclassified samples, which explains the limited performance observed in classifying NSCLC subtypes using mutational data and questions the current NSCLC subtype classification to adequately represent inherent mutational heterogeneity. This observation is of particular importance as specific mutational patterns in NSCLC have also been linked to clinical heterogeneity^21^. For instance, in non-squamous NSCLC, KRAS mutation has been shown to interact with co-occurring mutations in TP53, STK11, PTPRD, RBM10, and ATM with respect to immune checkpoint inhibitor efficacy^22^. Most of such interactions originate from tumor-initiating mutations in KRAS, TP53, and EGFR^23^, resulting in exclusivity patterns that have been found to be associated with response to both targeted^24^ and immunotherapy^25^.

Interestingly, the separation of Kaplan–Meier curves of progression-free survival (PFS) for LUAD vs. LUSC samples (Supplementary Fig. S3a) is also reflected in our low uncertainty predictions (Supplementary Fig. S3b). In contrast, high uncertainty predictions identified a subgroup of LUAD and LUSC samples which do not separate equally with respect to PFS (Supplementary Fig. S3c). As a result, our prediction uncertainty estimate may provide a mean to identify patients for whom the general prognostic trend does not apply.

Given the excellent classification accuracy of the expression-based profiling model, NSCLC histology seems to be associated to a greater extent with transcriptomic features rather than mutational profiles. Since expression-based regularization did not improve NSCLC subtype classification performance, this discriminatory power could not be transferred to clinically more available genomic data. However, substructures of the expression-based manifold were captured in the mutation-based manifold, indicating general feasibility. Therefore, the regularization based on gene expression, or other ’omics, data might prove beneficial in other clinically relevant prediction tasks.

While the expression-based profiling model achieves comparable classification performance to the histopathology-based assessment of NSCLC subtypes^26^, expression data is not widely available in clinical routine. In contrast, the classification performance of histopathology-based methods could not be achieved with a model based on clinically more available genomic data. However, we anticipate beneficial clinical utility if the comprehensive model, considering the mutational status of all genes, can be distilled to a reduced model, considering the mutational status of only a subset of genes, while preserving discriminatory power. In fact, the classification performance of a reduced model, comprising the 20 most important genes identified in our SHAP analysis, is comparable to that of the comprehensive model (Supplementary Fig. S4). Importantly, such a reduced gene panel might ultimately allow the non-invasive, and possibly earlier, histological assessment of NSCLC from a liquid biopsy.

As input to our model, we used a binary encoding of the mutational status of each gene. However, more details on the functional impact could be used, e.g., by separately considering low, medium, and high impact mutations, as obtained from Variant Effect Predictor (VEP)^27^. Furthermore, summary statistics could be constrained to mutational hotspots or gene regions encoding structural domains. However, both approaches may increase sparsity. Alternatively, protein language models have been described as zero-shot predictors of the functional effects of mutations^28^ and may be employed to encode missense mutations as a continuous metric. In addition, allele frequencies may need to be considered to improve the classification of samples of mixed subtype.

To deal with the curse of dimensionality, where the variance between samples becomes large and sparse^4^, the integration of prior knowledge about direct^29^ or indirect^30^ protein–protein interactions as relational inductive bias may help to effectively reduce the parameter space relative to the naive approach of modeling all interaction terms and, thus, may allow robust training of complex models on small cohorts.

Since our expression-aware genomic profiling model captures mutational patterns linked to histology and treatment efficacy, we anticipate our model to be suited as foundational model of genomic data for clinically relevant prognostic or predictive downstream tasks. To enhance its generalizability, regularization based on additional modalities and tasks may need to be integrated to enable learning a more holistic representation of the phenotype, which emanates from variation across all ’omics levels^31^.

## Materials and methods

### Data

The results of our work are based upon data generated by the TCGA Research Network: https://www.cancer.gov/tcga. The derived Pan-Cancer Atlas datasets^9^ were downloaded from cBioPortal^32^. We only included primary tumor samples of AD and SCC histology for which both mutation and expression data were available. Furthermore, we restricted our analysis to genes annotated in the Cancer Gene Census (CGC) of the Catalogue of Somatic Mutations In Cancer (COSMIC, version 95)^33^, excluding genes for which either mutation or expression data was not available, yielding 713 genes. Finally, we removed samples with incomplete data for the selected genes.

In total, our dataset comprised 1525 AD samples of 7 cancer types and 1233 SCC samples of 3 cancer types, including 992 (36%) NSCLC subtypes composed of 510 LUAD and 482 LUSC samples. Further cancer subtypes comprised prostate AC (n = 460), colon AC (n = 233), pancreatic AC (n = 172), rectal AC (n = 89), mucinous AC of the colon and rectum (n = 37), and endocervical AC (n = 24), as well as head and neck SCC (n = 507) and cervical SCC (n = 244). Progression-free survival analyses were conducted on a subset of 497 LUAD and 476 LUSC samples for which progression-free status annotations were available.

The gene mutational status was calculated based on the presence/absence of one or more non-synonymous mutations as determined with the Variant Effect Predictor (VEP)^27^. Gene expression values were based on RSEM z-scores.

### Methodology

**Figure 8:**
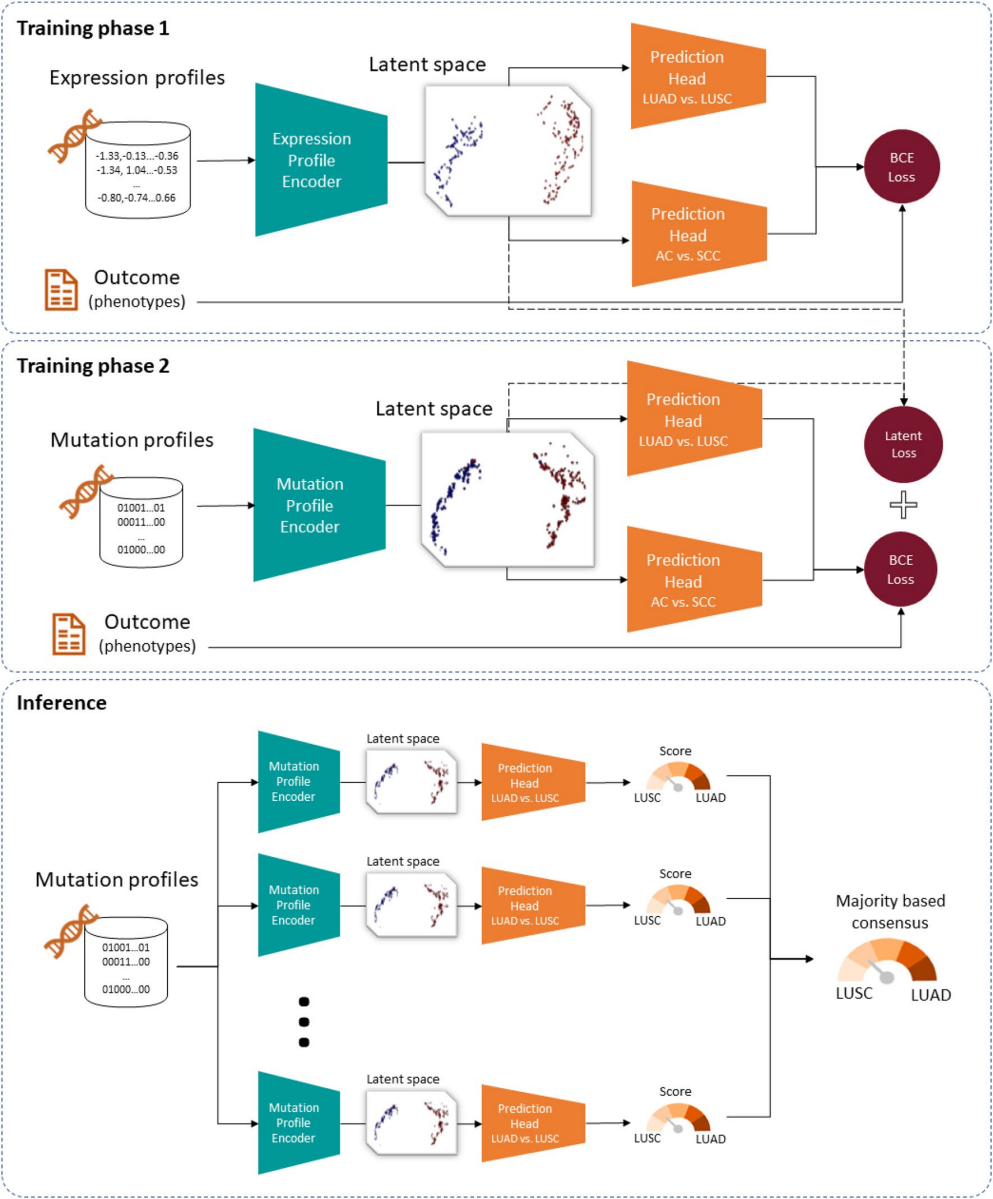
Architecture and training setup of our ensemble of expression-aware genomic profiling models.

Figure 8 shows an overview of the architecture and training setup of our model. The basic architecture consists of a multi-layer perceptron (MLP) with one or two prediction heads; one for classifying AC vs. SCC of the lung, and another for classifying AC vs. SCC irrespective of tissue type. The encoder is composed of three hidden layers with 256, 128, and 64 neurons, respectively. Each prediction head connects a dropout layer to its respective output neuron, using a dropout rate of 0.2. We employed rectified linear units (ReLUs) as activation functions of all hidden units and sigmoid activation functions at the output neurons.

Model training comprised two phases. First, a gene expression-based model (termed expression-based profiling model) was trained to classify samples into NSCLC subtypes by minimizing the sum of two binary cross entropy (BCE) loss terms (one for each prediction head). Subsequently, a gene mutation-based model (termed expression-aware genomic profiling model) was trained on the same classification task by adding to the loss an L2 regularization term that is calculated between the latent representation of the mutation-based model and the latent representation of the expression-based model.

Due to the small number of available samples, all experiments were conducted using a stratified tenfold cross-validation scheme. While, in each iteration, one fold was held back as testing set, the samples of the remaining nine folds were further partitioned into a training (80%) and a validation set (20%), again stratified with respect to NSCLC subtype. To train an ensemble of one hundred models, the training set was bootstrapped a hundred times, resulting in one hundred bootstrap samples (each serving as training set for one model). The bootstrap samples were the same size as the training set. The prediction score of the ensemble of models was obtained by averaging prediction values of all models predicting the majority predicted NSCLC subtype. To aggregate the results across testing folds, we averaged the true and false positive rates at each possible operating point in the ROC curve and calculated the AUC afterwards. Prediction uncertainties were calculated as the fraction of models predicting the majority predicted NSCLC subtype in all models of the ensemble (termed majority size).

The models were trained using the Adam optimizer with an initial learning rate of $10^{-4}$ and a batch size of 64. To counteract potential class imbalance effects, we sampled each training batch using a weighted random sampler^34^. Within each cross-validation iteration, the best model was selected as the one with minimum loss on the validation set, using a patience of 20 epochs. To enable optimal knowledge transfer from the expression-based profiling model, the expression-aware genomic profiling model was trained using only the L2 regularization term for 30 epochs (empirically determined from learning curves), before switching on the task-specific loss terms.

To visualize and compare the manifolds learned by different models, we conducted a non-parametric UMAP analysis^35^. The UMAP model was fit to the 64-dimensional latent representation obtained by applying the expression-based encoder to the testing set, with a local neighborhood of size 15. Subsequently, the UMAP model was used to transform the latent representation obtained by applying the genomic profiling model, trained with or without expression-based regularization, to the testing set.

We conducted a KernelSHAP^19^ analysis to estimate the average contribution of the mutational status of each gene to either model predictions or prediction uncertainties of different coalitions in comparison to the average prediction across all samples. Shapely values were separately estimated for each testing set and merged afterwards.

To create NSCLC subgroups based on the presence of a mutated LUAD driver gene, a mutated LUSC driver gene, or both (mixed driver mutations), we selected the most confident driver genes in either LUAD (BRAF, EGFR, KRAS, STK11) or LUSC (CDKN2A, NFE2L2, PIK3CA, PTEN) based on a Pan-Cancer Atlas analysis^36^ using a consensus score greater than four. TP53, which is a recurrently mutated driver gene in both subtypes, was excluded.

### Supplementary Information

Supplementary Information.

## Data Availability

The results shown here are based upon open access Pan-Cancer Atlas datasets^9^, derived from data generated by the TCGA Research Network (https://www.cancer.gov/tcga). The datasets are available for download on cBioPortal^32^ at https://cbioportal-datahub.s3.amazonaws.com/luad_tcga_pan_can_atlas_2018.tar.gz (LUAD), https://cbioportal-datahub.s3.amazonaws.com/lusc_tcga_pan_can_atlas_2018.tar.gz (LUSC), https://cbioportal-datahub.s3.amazonaws.com/prad_tcga_pan_can_atlas_2018.tar.gz (prostate AC), https://cbioportal-datahub.s3.amazonaws.com/coadread_tcga_pan_can_atlas_2018.tar.gz (colon AC, rectal AC, mucinous AC of the colon and rectum), https://cbioportal-datahub.s3.amazonaws.com/paad_tcga_pan_can_atlas_2018.tar.gz (pancreatic AC), https://cbioportal-datahub.s3.amazonaws.com/cesc_tcga_pan_can_atlas_2018.tar.gz (endocervical AC, cervical SCC), and https://cbioportal-datahub.s3.amazonaws.com/hnsc_tcga_pan_can_atlas_2018.tar.gz (head and neck SCC).

## Code availability

The code to perform inference of the models presented in this paper is available from the authors upon request.
