# Supplementary material for: Prediction uncertainty estimates elucidate the limitation of current NSCLC subtype classification in representing mutational heterogeneity
Source: Sci Rep. 2024 Mar 21;14:6779. doi: 10.1038/s41598-024-57057-3 (PMC10958018; doi:10.1038/s41598-024-57057-3)
Supplement: Supplementary file 1 — Supplementary Information. [file 41598_2024_57057_MOESM1_ESM.pdf]

Prediction uncertainty estimates elucidate the limitation of current NSCLC subtype classification in representing mutational heterogeneity

Andrei Puiu[1,2], Carlos Gómez Tapia[3], Maximilian Weiss[3], Vivek Singh[4], Ali Kamen[4], and Matthias Siebert[3,*]

[1]Siemens SRL, Advanta, Brasov, 500007, Romania
[2]Transilvania University of Brasov, Automation and Information Technology, Brasov, 500174, Romania
[3]Siemens Healthineers, Digital Technology and Innovation, Erlangen, 91052, Germany
[4]Siemens Healthineers, Digital Technology and Innovation, Princeton, 08540, USA
[*]matthias.siebert@siemens-healthineers.com

Supplementary Figures

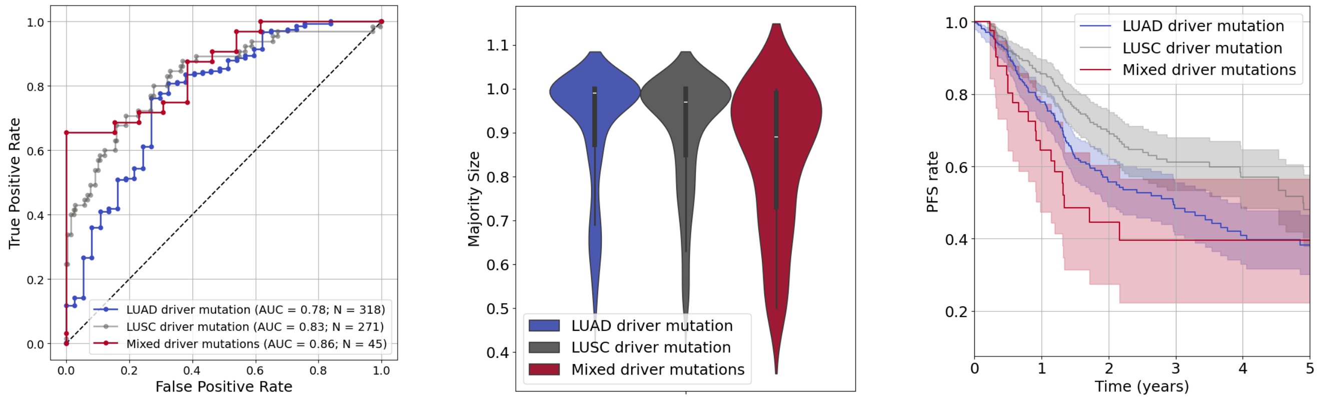

(a) **(b)** **(c)**

Supplementary Figure S1. Comparative analysis of NSCLC subgroups based on the presence of a mutated LUAD driver gene (BRAF, EGFR, KRAS, or STK11), a mutated LUSC driver gene (CDKN2A, NFE2L2, PIK3CA, or PTEN), or both (mixed driver mutations). (a) Performance of the ensemble of expression-aware genomic profiling models, trained to classify NSCLC subtypes using mutational data of the extended dataset, across subgroups. The dashed line represents the ROC curve of a random classifier. (b) Distribution of the prediction uncertainty estimate (majority size) across subgroups. (c) Kaplan-Meier curves of progression-free survival (PFS) across subgroups.

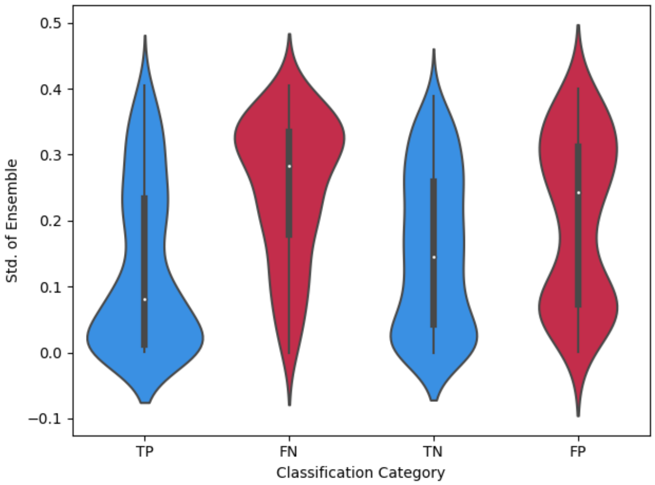

Supplementary Figure S2. Distribution of the standard deviation (std.) of ensemble predictions as uncertainty estimate for different classification categories, with TP, FN, TN, and FP corresponding to true positive, false negative, true negative, and false positive predictions, respectively. The ensemble of expression-aware genomic profiling models was trained to classify NSCLC subtypes using mutational data of the extended dataset.

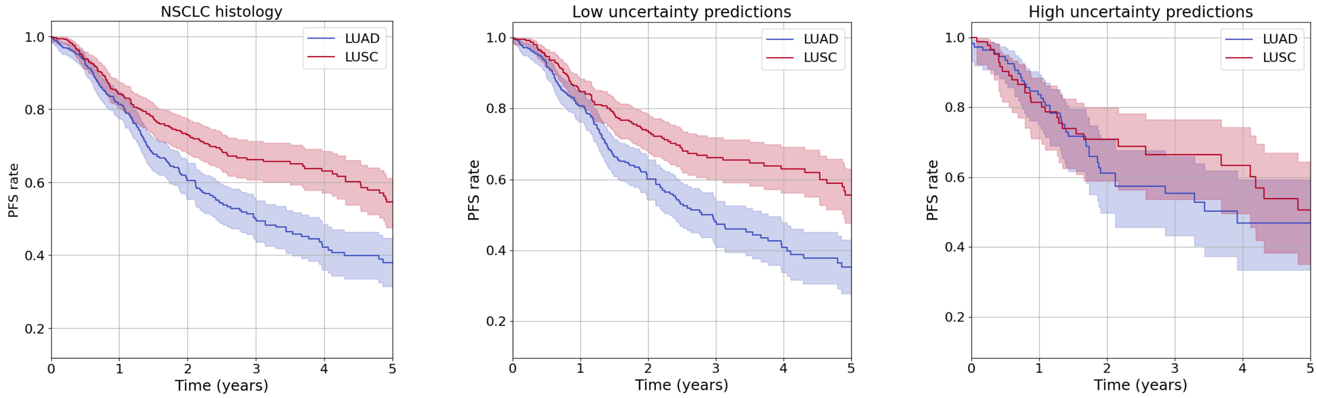

(a) (b) (c)

Supplementary Figure S3. Kaplan-Meier curves of progression-free survival (PFS) for NSCLC samples, separated by (a) histology as well as (b) low uncertainty and (c) high-uncertainty subtype predictions of the ensemble of expression-aware genomic profiling models trained to classify NSCLC subtypes using mutational data of the extended dataset, respectively.

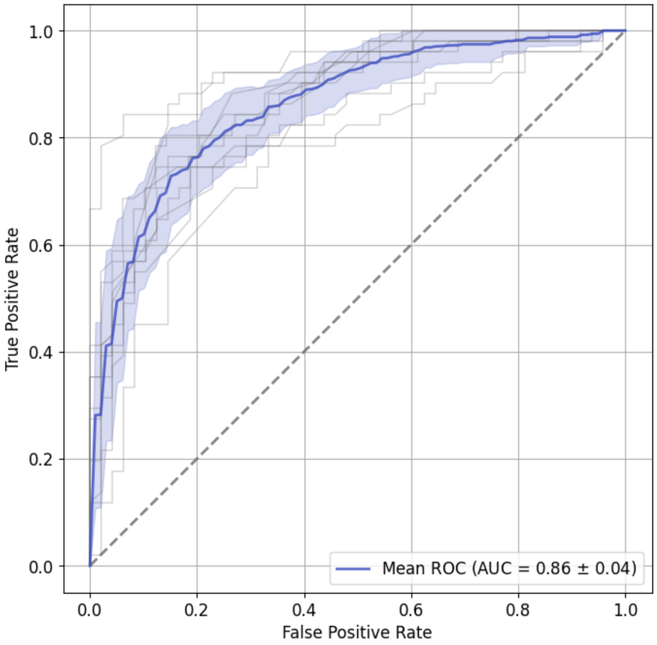

Supplementary Figure S4. Performance of the distilled genomic profiling model, comprising the 20 most important genes identified in the cohort-level SHAP analysis reported in Figure 5a, to classify NSCLC subtypes using mutational data. The blue line and light blue area depict the mean ROC curve and its standard deviation, respectively, across ten cross-validation folds. Grey lines correspond to ROC curves of individual folds. The dashed line represents the ROC curve of a random classifier.

Supplementary Tables

NSCLC subgroup based on mutated gene				NSCLC subgroup based on histology		
**Gene**	**N**	**Univar. acc. [%]**	**Model acc. [%]**	**Histology**	**Univar. acc. [%]**	**Model acc. [%]**
KRAS	161	**95.7**	88.8	LUAD	30.2	**72.2**
TP53	650	60.8	**74.0**	LUSC	**81.6**	77.1
EGFR	77	84.4	**85.7**	LUAD	12.7	**72.2**
NFE2L2	86	83.7	**86.0**	LUSC	14.9	**77.1**
FAM135B	193	60.6	**74.1**	LUSC	24.2	**77.1**
KMT2D	147	74.1	**82.3**	LUSC	22.5	**77.1**
FAT1	119	56.3	**73.1**	LUSC	13.8	**77.1**
PIK3CA	81	65.4	**75.3**	LUSC	11.0	**77.1**
STK11	77	**93.5**	80.5	LUAD	14.1	**72.2**
CDKN2A	91	78	**83.5**	LUSC	14.7	**77.1**
APC	52	48.1	**69.2**	LUAD	4.9	**72.2**
NOTCH1	62	61.3	**82.3**	LUSC	7.9	**77.1**
BRAF	56	73.2	**76.8**	LUAD	8.0	**72.2**
LRP1B	347	49.6	**72.0**	LUSC	35.5	**77.1**
CSMD3	409	50.1	**74.3**	LUSC	42.4	**77.1**
PTEN	57	87.7	**89.5**	LUSC	10.3	**77.1**
PTPRD	115	**73.0**	69.6	LUAD	16.5	**72.2**
ROBO2	66	**71.2**	65.2	LUAD	9.2	**72.2**
ATM	70	60	**78.6**	LUAD	8.2	**72.2**
PTPRB	73	57.5	**75.3**	LUSC	8.7	**77.1**

Supplementary Table S1. Comparison of the classification accuracy (acc.) between univariable models (Univar.) and our ensemble of expression-aware genomic profiling models trained to classify NSCLC subtypes using mutational data of the extended dataset (Model), on different NSCLC subgroups. Left: NSCLC subgroups are based on the presence of a non-synonymous mutation in the respective gene, and are listed with the number of samples included (N). Right: NSCLC subgroups correspond to the histological subtype the mutated gene is mostly associated with. In the presence of the mutated gene, the univariable model assigns the sample to the NSCLC subtype the mutated gene is mostly associated with, and to the other NSCLC subtype, otherwise. Its accuracy corresponds to the fraction of LUAD samples in all samples carrying the mutated gene (left) and the overall fraction of LUAD samples carrying the mutated gene (right), respectively. Genes were selected in accordance with the cohort-level SHAP summary provided in Figure 5a.

Gene	NSCLC [%]	LUAD [%]	LUSC [%]
KRAS	16.2	30.2	1.4
TP53	65.4	50.0	81.6
EGFR	7.7	12.7	2.5
NFE2L2	8.7	2.7	14.9
FAM135B	19.4	14.9	24.2
KMT2D	14.8	7.5	22.5
FAT1	12.0	10.2	13.8
PIK3CA	8.1	5.5	11.0
STK11	7.7	14.1	1.0
CDKN2A	9.2	3.9	14.7
APC	5.2	4.9	5.6
NOTCH1	6.2	4.7	7.9
BRAF	5.6	8.0	3.1
LRP1B	34.9	34.3	35.5
CSMD3	41.1	40.0	42.4
PTEN	5.7	1.4	10.3
PTPRD	11.6	16.5	6.4
ROBO2	6.6	9.2	3.9
ATM	7.0	8.2	5.8
PTPRB	7.3	6.1	8.7

Supplementary Table S2. Frequency of NSCLC, LUAD, and LUSC samples carrying a non-synonymous mutation in the respective gene. Genes were selected in accordance with the cohort-level SHAP summary provided in Figure 5a.
